# 2-(2-Methyl-6-phenyl-1-propyl-1,4-dihydro­pyridin-4-yl­idene)propane­dinitrile

**DOI:** 10.1107/S1600536811005587

**Published:** 2011-02-19

**Authors:** Young Hyun Kim, Hyung Jin Kim, Enkhzul Otgonbaatar, Chee-Hun Kwak

**Affiliations:** a School of Applied Chemical Engineering, Chonnam National University, Gwangju 500-757, Republic of Korea; b Department of Chemistry, Sunchon National University, 315 Maegok Dong, Sunchon, Jeonnam 540-742, Republic of Korea

## Abstract

In the title compound, C_18_H_17_N_3_, the dihedral angle between the dihydropyridine and phenyl rings is 72.57 (5)° and that between the dihydropyridine ring and malononitrile plane is 5.19 (20)°. The C—C bond lengths in the pyridine ring are considerably shorter than those of normal single bonds, indicating that electrons on the dihydropyridine ring, including the non-bonding electrons of the N atom, are delocalized on the ring.

## Related literature

For the synthesis of the starting material, see: Tolmachev *et al.* (2006 ▶). For a related structure, see: Ha *et al.* (2009 ▶).
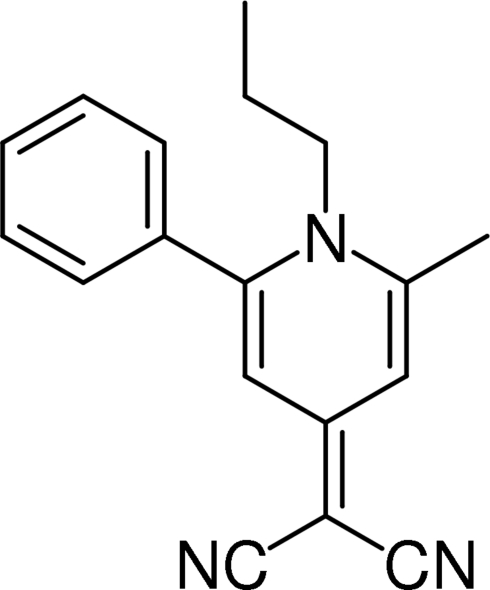


## Experimental

### 

#### Crystal data

C_18_H_17_N_3_
*M* *_r_* = 275.35
Monoclinic, 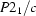

*a* = 11.5580 (7) Å
*b* = 9.9179 (6) Å
*c* = 13.9268 (7) Å
β = 105.707 (2)°
*V* = 1536.83 (15) Å^3^
*Z* = 4
Mo *K*α radiation
μ = 0.07 mm^−1^
*T* = 100 K
0.5 × 0.4 × 0.2 mm

#### Data collection

Rigaku R-AXIS RAPID II-S diffractometer
Absorption correction: multi-scan (*RAPID-AUTO*; Rigaku, 2008 ▶) *T*_min_ = 0.966, *T*_max_ = 0.986
13505 measured reflections
3185 independent reflections
2321 reflections with *I* > 2σ(*I*)
*R*_int_ = 0.077

#### Refinement

*R*[*F* ^2^ > 2σ(*F* ^2^)] = 0.049
*wR*(*F* ^2^) = 0.134
*S* = 1.07
3185 reflections
193 parameters
H-atom parameters constrained
Δρ_max_ = 0.22 e Å^−3^
Δρ_min_ = −0.20 e Å^−3^

### 

Data collection: *RAPID-AUTO* (Rigaku, 2008 ▶); cell refinement: *RAPID-AUTO*; data reduction: *RAPID-AUTO*; program(s) used to solve structure: *SHELXS97* (Sheldrick, 2008 ▶); program(s) used to refine structure: *SHELXL97* (Sheldrick, 2008 ▶); molecular graphics: *ORTEP-3* (Farrugia, 1997 ▶); software used to prepare material for publication: *WinGX* (Farrugia, 1999 ▶).

## Supplementary Material

Crystal structure: contains datablocks I, global. DOI: 10.1107/S1600536811005587/bq2278sup1.cif

Structure factors: contains datablocks I. DOI: 10.1107/S1600536811005587/bq2278Isup2.hkl

Additional supplementary materials:  crystallographic information; 3D view; checkCIF report

## Figures and Tables

**Fig. 1.**
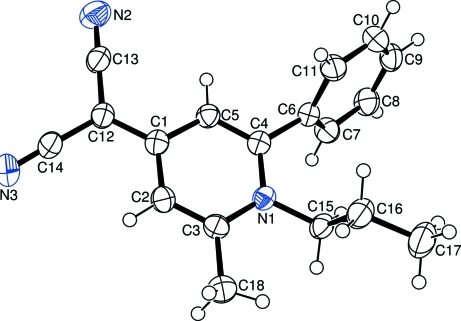
The structure of the title compound with displacement ellipsoids drawn at the 50% probability level for non-H atoms.

## supplementary crystallographic information

### Comment

Recently we have reported the structure of 2-(1-propyl-2,6-distyryl-1,4-pyridin-4-ylidene)malononitrile as a fluorescent dye (Ha *et al.*, 2009). Continuing our study on the (1,4-pyridin-4-ylidene)malononitrile derivatives, the title compound was synthesized and its structure was confirmed by ^1^H NMR and X-ray crystal analysis.

In the title compound, C_18_H_17_N_3_, the dihedral angles between the central pyridine and phenyl ring is 72.57 (5)° and that between the pyridine ring and malonitrile plane (N2 C13 C12 C14 N3 plane) is 5.19 (20)°. The bond distances of C—C bonds in the pyridine ring are considerably shorter than those of normal single bonds (D(C1—C2) = D(C1—C5) = 1.413 (3) Å). These results suggest that the electrons on the pyridine ring including non-bonding electrons of N1 are delocalized on the ring (Fig. 1).

### Experimental

A mixture of 2-(2-methyl-6-phenyl-4*H*-pyran-4-ylidene)malononitrile (1.5 g, 6.4 mmol) and *n*-propylamine (20 ml) was heated at 150 °C for 3 h. The mixture was cooled and concentrated under vacuum. Crude product was recrystallized from MeOH to give crystals suitable for X-ray analysis (1.20 g, 68%). Mp 166–167 °C. ^1^H NMR (300 MHz, CDCl_3_) δ 7.52–7.26 (m, 5H, Ph), 6.79 (d, 1H, J = 2.5 Hz, C—C*H*=C—N), 6.70 (d, 1H, J = 2.5 Hz), 3.75 (t, 2H, J = 8.1 Hz, NC*H*_2_CH_2_CH_3_), 2.50 (s, 3H, C*H*_3_), 1.52 (m, 2H, NCH_2_C*H*_2_CH_3_), 0.70 (t, 3H, J = 7.4 Hz, NCH_2_CH_2_C*H*_3_)

### Refinement

H atoms were positioned geometrically and allowed to ride on their respective parent atoms [C—H = 0.93 (CH, *sp*^2^), 0.96 (CH_3_), 0.97Å (CH_2_), respectively and *U*_iso_(H) = 1.2*U*_eq_(C).

### Crystal data

**Table:**

C_18_H_17_N_3_	*F*(000) = 584
*M**_r_* = 275.35	Z = 4
Monoclinic, *P*2_1_/*c*	*D*_x_ = 1.190 Mg m^−^^3^
Hall symbol: -P 2ybc	Mo *K*α radiation, λ = 0.71073 Å
*a* = 11.5580 (7) Å	Cell parameters from 15051 reflections
*b* = 9.9179 (6) Å	θ = 27.5–3.0°
*c* = 13.9268 (7) Å	µ = 0.07 mm^−^^1^
β = 105.707 (2)°	*T* = 100 K
*V* = 1536.83 (15) Å^3^	Block, yellow
*Z* = 4	0.5 × 0.4 × 0.2 mm

### Data collection

**Table:**

Rigaku R-AXIS RAPID II-S diffractometer	3185 independent reflections
Radiation source: fine-focus sealed tube	2321 reflections with *I* > 2σ(*I*)
graphite	*R*_int_ = 0.077
ω scans	θ_max_ = 26.5°, θ_min_ = 3.0°
Absorption correction: multi-scan (*RAPID-AUTO*; Rigaku, 2008)	*h* = −14→14
*T*_min_ = 0.966, *T*_max_ = 0.986	*k* = −12→12
13505 measured reflections	*l* = −16→17

### Refinement

**Table:**

Refinement on *F*^2^	Secondary atom site location: difference Fourier map
Least-squares matrix: full	Hydrogen site location: inferred from neighbouring sites
*R*[*F*^2^ > 2σ(*F*^2^)] = 0.049	H-atom parameters constrained
*wR*(*F*^2^) = 0.134	*w* = 1/[σ^2^(*F*_o_^2^) + (0.0522*P*)^2^ + 0.3213*P*] where *P* = (*F*_o_^2^ + 2*F*_c_^2^)/3
*S* = 1.07	(Δ/σ)_max_ < 0.001
3185 reflections	Δρ_max_ = 0.22 e Å^−^^3^
193 parameters	Δρ_min_ = −0.20 e Å^−^^3^
0 restraints	Extinction correction: *SHELXL97* (Sheldrick, 2008), Fc^*^=kFc[1+0.001xFc^2^λ^3^/sin(2θ)]^-1/4^
Primary atom site location: structure-invariant direct methods	Extinction coefficient: 0.013 (3)

### Special details

**Table:**

Geometry. All e.s.d.'s (except the e.s.d. in the dihedral angle between two l.s. planes) are estimated using the full covariance matrix. The cell e.s.d.'s are taken into account individually in the estimation of e.s.d.'s in distances, angles and torsion angles; correlations between e.s.d.'s in cell parameters are only used when they are defined by crystal symmetry. An approximate (isotropic) treatment of cell e.s.d.'s is used for estimating e.s.d.'s involving l.s. planes.
Refinement. Refinement of *F*^2^ against ALL reflections. The weighted *R*-factor *wR* and goodness of fit *S* are based on *F*^2^, conventional *R*-factors *R* are based on *F*, with *F* set to zero for negative *F*^2^. The threshold expression of *F*^2^ > σ(*F*^2^) is used only for calculating *R*-factors(gt) *etc*. and is not relevant to the choice of reflections for refinement. *R*-factors based on *F*^2^ are statistically about twice as large as those based on *F*, and *R*- factors based on ALL data will be even larger.

### Fractional atomic coordinates and isotropic or equivalent isotropic displacement parameters (Å^2^)

**Table:**

	*x*	*y*	*z*	*U*_iso_*/*U*_eq_	
C1	0.86513 (14)	0.37654 (15)	0.41005 (10)	0.0322 (4)	
C2	0.94627 (14)	0.43142 (16)	0.36078 (11)	0.0350 (4)	
H2	1.0277	0.4120	0.3851	0.042*	
C3	0.90918 (14)	0.51234 (16)	0.27834 (11)	0.0345 (4)	
C4	0.70719 (14)	0.49082 (15)	0.28609 (10)	0.0311 (4)	
C5	0.74344 (14)	0.41302 (16)	0.36931 (11)	0.0332 (4)	
H5	0.6864	0.3829	0.4004	0.040*	
C6	0.57636 (14)	0.51915 (15)	0.24329 (11)	0.0325 (4)	
C7	0.51068 (15)	0.45459 (18)	0.15657 (12)	0.0402 (4)	
H7	0.5498	0.3993	0.1214	0.048*	
C8	0.38795 (16)	0.4724 (2)	0.12290 (13)	0.0466 (4)	
H8	0.3443	0.4281	0.0657	0.056*	
C9	0.32989 (16)	0.55609 (19)	0.17404 (13)	0.0467 (5)	
H9	0.2473	0.5686	0.1506	0.056*	
C10	0.39303 (17)	0.62090 (19)	0.25902 (14)	0.0478 (5)	
H10	0.3534	0.6773	0.2931	0.057*	
C11	0.51660 (15)	0.60193 (18)	0.29407 (12)	0.0409 (4)	
H11	0.5594	0.6452	0.3521	0.049*	
C12	0.90262 (14)	0.28936 (16)	0.49335 (11)	0.0357 (4)	
C13	0.82087 (16)	0.24072 (17)	0.54461 (12)	0.0399 (4)	
C14	1.02361 (17)	0.24658 (18)	0.52802 (12)	0.0436 (4)	
C15	0.74831 (15)	0.63071 (16)	0.15180 (11)	0.0366 (4)	
H15A	0.6704	0.5997	0.1122	0.044*	
H15B	0.8041	0.6235	0.1110	0.044*	
C16	0.73834 (19)	0.77697 (18)	0.17897 (12)	0.0481 (5)	
H16A	0.8174	0.8115	0.2129	0.058*	
H16B	0.6877	0.7844	0.2241	0.058*	
C17	0.6847 (2)	0.8605 (2)	0.08532 (15)	0.0669 (6)	
H17A	0.6826	0.9537	0.1032	0.100*	
H17B	0.6045	0.8298	0.0542	0.100*	
H17C	0.7333	0.8503	0.0397	0.100*	
C18	0.99893 (17)	0.5697 (2)	0.22903 (14)	0.0500 (5)	
H18A	0.9884	0.6655	0.2224	0.075*	
H18B	0.9869	0.5300	0.1642	0.075*	
H18C	1.0788	0.5500	0.2690	0.075*	
N1	0.78979 (11)	0.54168 (13)	0.24021 (9)	0.0320 (3)	
N2	0.75433 (16)	0.20179 (18)	0.58685 (12)	0.0569 (5)	
N3	1.12223 (16)	0.2122 (2)	0.55469 (12)	0.0672 (5)	

### Atomic displacement parameters (Å^2^)

**Table:**

	*U*^11^	*U*^22^	*U*^33^	*U*^12^	*U*^13^	*U*^23^
C1	0.0369 (9)	0.0294 (8)	0.0286 (7)	−0.0009 (7)	0.0057 (6)	−0.0046 (6)
C2	0.0321 (8)	0.0349 (9)	0.0361 (8)	0.0006 (7)	0.0058 (6)	−0.0010 (6)
C3	0.0334 (9)	0.0337 (9)	0.0367 (8)	−0.0020 (7)	0.0100 (6)	−0.0031 (6)
C4	0.0342 (9)	0.0282 (8)	0.0299 (7)	−0.0015 (6)	0.0069 (6)	−0.0038 (6)
C5	0.0332 (8)	0.0354 (8)	0.0303 (8)	−0.0013 (7)	0.0073 (6)	−0.0003 (6)
C6	0.0333 (8)	0.0324 (8)	0.0306 (7)	0.0024 (7)	0.0068 (6)	0.0039 (6)
C7	0.0382 (9)	0.0434 (10)	0.0372 (8)	0.0010 (7)	0.0070 (7)	−0.0041 (7)
C8	0.0399 (10)	0.0557 (11)	0.0386 (9)	−0.0008 (8)	0.0012 (7)	0.0013 (8)
C9	0.0350 (10)	0.0525 (11)	0.0496 (10)	0.0065 (8)	0.0063 (8)	0.0144 (8)
C10	0.0476 (11)	0.0480 (11)	0.0518 (10)	0.0119 (9)	0.0204 (8)	0.0050 (8)
C11	0.0431 (10)	0.0425 (10)	0.0364 (8)	0.0032 (8)	0.0097 (7)	−0.0027 (7)
C12	0.0371 (9)	0.0361 (9)	0.0320 (8)	0.0022 (7)	0.0062 (6)	0.0017 (6)
C13	0.0469 (10)	0.0386 (9)	0.0326 (8)	0.0038 (8)	0.0081 (7)	0.0025 (7)
C14	0.0484 (11)	0.0502 (11)	0.0321 (8)	0.0099 (9)	0.0105 (7)	0.0071 (7)
C15	0.0431 (9)	0.0387 (9)	0.0276 (7)	0.0021 (7)	0.0091 (7)	0.0022 (6)
C16	0.0662 (13)	0.0414 (10)	0.0380 (9)	0.0068 (9)	0.0163 (8)	0.0044 (7)
C17	0.0989 (18)	0.0518 (12)	0.0534 (11)	0.0250 (12)	0.0265 (11)	0.0160 (9)
C18	0.0451 (11)	0.0524 (11)	0.0554 (11)	−0.0005 (8)	0.0185 (8)	0.0122 (9)
N1	0.0356 (7)	0.0310 (7)	0.0287 (6)	0.0000 (5)	0.0075 (5)	0.0001 (5)
N2	0.0670 (11)	0.0572 (11)	0.0521 (9)	0.0020 (9)	0.0258 (9)	0.0113 (8)
N3	0.0545 (11)	0.0899 (14)	0.0562 (10)	0.0296 (10)	0.0130 (8)	0.0229 (9)

### Geometric parameters (Å, °)

**Table:**

C1—C2	1.412 (2)	C10—H10	0.9300
C1—C5	1.414 (2)	C11—H11	0.9300
C1—C12	1.417 (2)	C12—C13	1.414 (2)
C2—C3	1.371 (2)	C12—C14	1.415 (2)
C2—H2	0.9300	C13—N2	1.154 (2)
C3—N1	1.369 (2)	C14—N3	1.151 (2)
C3—C18	1.502 (2)	C15—N1	1.4852 (18)
C4—C5	1.361 (2)	C15—C16	1.511 (2)
C4—N1	1.3801 (19)	C15—H15A	0.9700
C4—C6	1.494 (2)	C15—H15B	0.9700
C5—H5	0.9300	C16—C17	1.527 (2)
C6—C11	1.384 (2)	C16—H16A	0.9700
C6—C7	1.396 (2)	C16—H16B	0.9700
C7—C8	1.380 (2)	C17—H17A	0.9600
C7—H7	0.9300	C17—H17B	0.9600
C8—C9	1.380 (3)	C17—H17C	0.9600
C8—H8	0.9300	C18—H18A	0.9600
C9—C10	1.371 (3)	C18—H18B	0.9600
C9—H9	0.9300	C18—H18C	0.9600
C10—C11	1.391 (2)		
			
C2—C1—C5	115.20 (13)	C13—C12—C14	117.32 (14)
C2—C1—C12	122.45 (14)	C13—C12—C1	121.55 (14)
C5—C1—C12	122.34 (14)	C14—C12—C1	121.13 (15)
C3—C2—C1	122.29 (15)	N2—C13—C12	179.5 (2)
C3—C2—H2	118.9	N3—C14—C12	178.88 (18)
C1—C2—H2	118.9	N1—C15—C16	113.11 (12)
N1—C3—C2	120.21 (14)	N1—C15—H15A	109.0
N1—C3—C18	119.37 (14)	C16—C15—H15A	109.0
C2—C3—C18	120.42 (15)	N1—C15—H15B	109.0
C5—C4—N1	120.64 (14)	C16—C15—H15B	109.0
C5—C4—C6	119.44 (13)	H15A—C15—H15B	107.8
N1—C4—C6	119.92 (12)	C15—C16—C17	110.33 (14)
C4—C5—C1	122.03 (14)	C15—C16—H16A	109.6
C4—C5—H5	119.0	C17—C16—H16A	109.6
C1—C5—H5	119.0	C15—C16—H16B	109.6
C11—C6—C7	118.97 (15)	C17—C16—H16B	109.6
C11—C6—C4	119.92 (14)	H16A—C16—H16B	108.1
C7—C6—C4	120.90 (14)	C16—C17—H17A	109.5
C8—C7—C6	120.24 (16)	C16—C17—H17B	109.5
C8—C7—H7	119.9	H17A—C17—H17B	109.5
C6—C7—H7	119.9	C16—C17—H17C	109.5
C7—C8—C9	120.04 (16)	H17A—C17—H17C	109.5
C7—C8—H8	120.0	H17B—C17—H17C	109.5
C9—C8—H8	120.0	C3—C18—H18A	109.5
C10—C9—C8	120.53 (17)	C3—C18—H18B	109.5
C10—C9—H9	119.7	H18A—C18—H18B	109.5
C8—C9—H9	119.7	C3—C18—H18C	109.5
C9—C10—C11	119.70 (16)	H18A—C18—H18C	109.5
C9—C10—H10	120.1	H18B—C18—H18C	109.5
C11—C10—H10	120.1	C3—N1—C4	119.59 (12)
C6—C11—C10	120.52 (15)	C3—N1—C15	120.88 (13)
C6—C11—H11	119.7	C4—N1—C15	119.51 (13)
C10—C11—H11	119.7		
			
C5—C1—C2—C3	−1.1 (2)	C4—C6—C11—C10	−175.04 (15)
C12—C1—C2—C3	177.93 (14)	C9—C10—C11—C6	0.6 (3)
C1—C2—C3—N1	−0.6 (2)	C2—C1—C12—C13	176.88 (15)
C1—C2—C3—C18	179.35 (15)	C5—C1—C12—C13	−4.2 (2)
N1—C4—C5—C1	−2.5 (2)	C2—C1—C12—C14	−3.7 (2)
C6—C4—C5—C1	176.43 (14)	C5—C1—C12—C14	175.28 (15)
C2—C1—C5—C4	2.6 (2)	N1—C15—C16—C17	−174.92 (16)
C12—C1—C5—C4	−176.36 (14)	C2—C3—N1—C4	0.9 (2)
C5—C4—C6—C11	69.6 (2)	C18—C3—N1—C4	−179.09 (15)
N1—C4—C6—C11	−111.43 (17)	C2—C3—N1—C15	179.15 (14)
C5—C4—C6—C7	−105.08 (17)	C18—C3—N1—C15	−0.8 (2)
N1—C4—C6—C7	73.9 (2)	C5—C4—N1—C3	0.7 (2)
C11—C6—C7—C8	−0.6 (2)	C6—C4—N1—C3	−178.28 (13)
C4—C6—C7—C8	174.15 (15)	C5—C4—N1—C15	−177.62 (13)
C6—C7—C8—C9	1.1 (3)	C6—C4—N1—C15	3.4 (2)
C7—C8—C9—C10	−0.7 (3)	C16—C15—N1—C3	−93.10 (18)
C8—C9—C10—C11	−0.1 (3)	C16—C15—N1—C4	85.16 (18)
C7—C6—C11—C10	−0.2 (2)		
